# Epigenetic Regulation of Non-Lymphoid Cells by Bisphenol A, a Model Endocrine Disrupter: Potential Implications for Immunoregulation

**DOI:** 10.3389/fendo.2015.00091

**Published:** 2015-06-05

**Authors:** Deena Khan, S. Ansar Ahmed

**Affiliations:** ^1^Department of Biomedical Sciences and Pathobiology, Virginia-Maryland College of Veterinary Medicine, Virginia Tech, Blacksburg, VA, USA

**Keywords:** bisphenol A, EDC, immune, epigenetics, estrogenic

## Abstract

Endocrine disrupting chemicals (EDC) abound in the environment since many compounds are released from chemical, agricultural, pharmaceutical, and consumer product industries. Many of the EDCs such as Bisphenol A (BPA) have estrogenic activity or interfere with endogenous sex hormones. Experimental studies have reported a positive correlation of BPA with reproductive toxicity, altered growth, and immune dysregulation. Although the precise relevance of these studies to the environmental levels is unclear, nevertheless, their potential health implications remain a concern. One possible mechanism by which BPA can alter genes is by regulating epigenetics, including microRNA, alteration of methylation, and histone acetylation. There is now wealth of information on BPA effects on non-lymphoid cells and by comparison, paucity of data on effects of BPA on the immune system. In this mini review, we will highlight the BPA regulation of estrogen receptor-mediated immune cell functions and in different inflammatory conditions. In addition, BPA-mediated epigenetic regulation of non-lymphoid cells is emphasized. We recognize that most of these studies are on non-lymphoid cells, and given that BPA also affects the immune system, it is plausible that BPA could have similar epigenetic regulation in immune cells. It is hoped that this review will stimulate studies in this area to ascertain whether or not BPA epigenetically regulates the cells of the immune system.

## Introduction

Exposure to environmental chemicals is suspected in increase in the incidence of allergies and autoimmune diseases (1, 2). Among these compounds that are released in the environment, a group of compounds that alter the endocrine functions of body are termed as endocrine disrupting chemicals (EDCs). EDCs can interfere with synthesis, transport, function and activity, or elimination of natural hormones such as estrogen. This mini-review is focused on Bisphenol A (BPA; 2,2-bis (4-hydroxyphenyl) propane), a ubiquitous EDC, known to possess both agonistic and antagonistic estrogen action. It interferes with estrogen-regulated endocrine and physiological functions (3, 4). There is now growing evidence that BPA can alter epigenetics in various non-lymphoid cells. There is paucity of similar data on epigenetic regulation of BPA on the cells of the immune system. Given that BPA can modulate the immune system, it is plausible that the findings of BPA regulation of epigenetics in non-lymphoid cells may also apply to the immune system.

Bisphenol A, a xenoestrogen, is found in a variety of daily consumer products such as polycarbonate plastics, food can liners, epoxy resin, and flame retardant (5, 6). Nearly eight billion pounds of BPA is produced/year and more than 100 t is released in atmosphere (5). In 2003–2004 National Health and Nutrition Examination Survey, around 92.6% of 2517 subjects had detectable levels of BPA in their urine (7). In addition, BPA is also detected in sera, amniotic fluid, placenta, umbilical cord blood, ovarian follicular fluid, and colostrum (8–10). Although BPA interacts with both estrogen receptors (ERα and ERβ), BPA has 10 times higher affinity for ERβ (11, 12). Interestingly, BPA estrogenic metabolite, 4-Methyl-2,4-bis(4-hydroxyphenyl)pent-1-ene is more potent (∼500 fold) (13, 14). BPA exposure in different experimentally and naturally exposed populations during early developmental stages has been associated with reproductive abnormalities such as infertility in both males and females, altered male sexual function, spermatogenesis, endometrial disorders, polycystic ovary syndrome, interference of embryonic development programs, sex differentiation of the brain and behavior, metabolic disorders, and immune responsiveness (15–22).

## BPA a Potent Immunomodulator

It is well established that estrogen is a natural target of the immune system since both ERα and ERβ are present on cells of the immune system (23–25). Extensive studies have documented the immunomodulatory role of estrogen (23, 26–31). Increasing evidence suggests that BPA also modulate immune pathways, which may contribute to the development of inflammatory conditions and autoimmune diseases (1, 2, 32). BPA exposure modulates estrogen-associated immune signaling, molecular mimicry (33), disruption of cytochrome p450 enzyme (34), alteration of immune signaling in cells of innate and adaptive immune system, cytokine polarization to Th1 and Th2 (35), inhibition of Tregs (36), dysregulation of immunoglobulin (37), and hyperprolactinemia reviewed in detail previously (38, 39).

### BPA binds to ER to modulate immune cell signaling and function

Bisphenol A binds and stimulates ERα and ERβ transcriptional activity at concentrations of 100–1000 nM (12). However, BPA potency is 10–1000-fold less than other EDCs such as diethylstilbestrol (DES) and ethinyl estradiol (40). BPA treatment had opposing effects on ERα expression with decreased expression in males and increased expression in females F(0) and F(1) offspring rats (41). In a proteome study, apo-AI, DPPIII, and VAT were identified as protein biomarkers for BPA-induced endocrine disruption in spleen and thymus from mice prenatally exposed to BPA (42). Interestingly, female and young offsprings were more susceptible to alterations in these proteins suggesting gender and age interplay in BPA (42). Furthermore, there was decreased IL-2, IL-12, IFNγ, and TNFα expression in the spleen of BPA-treated rats when compared to control rats (41).

**Figure 1:**
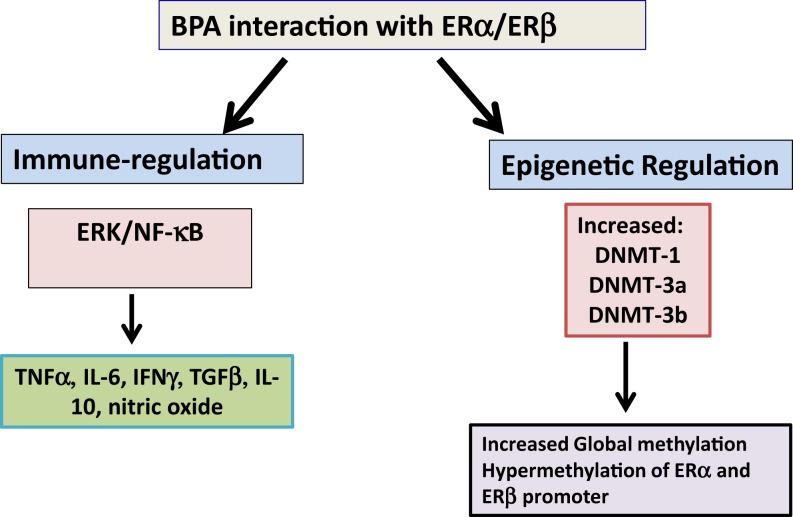
**Bisphenol A interaction with estrogen receptors**. Bisphenol A (BPA) interacts with estrogen receptor (ER)-α and β to regulate different proinflammatory cytokines such as TNFα, IL-6, and IFNγ and anti-inflammatory cytokines TGFβ and IL-10. In addition, it upregulates DNA methyl transferase enzymes (DNMTs) to epigenetically regulate gene expression. Abbreviation: IL: interleukin; ERK: extracellular signal-regulated kinases.

It has been recently shown that treatment of BV2 murine microglial cell line, THP1 macrophage and primary human macrophages with BPA increased TNFα and IL-6 but decreased IL-10 and TGFβ (Figure 1) (43, 44). This was mediated through ERα/β and extracellular-regulated protein kinases (ERK)/nuclear factor κB (NF-κB) signal cascade (43, 44). Interestingly, treatment of LPS-activated RAW 264.7 cells and murine macrophages with BPA decreased nitric oxide production (45–47). These effects are potentially mediated through BPA-ER mediated downregulation of NF-κB transactivation (48). However, BPA-exposed zebra fish embryos have increased expression of iNOS, IFNγ, IL-1β, IL-10, TNFα, CC-chemokine, and CXCL-clc. In addition, BPA altered expression of members of Toll-like receptors (TLRs) signaling pathway TLR3, TRIF, MyD88, IRAK4, and TRAF6 (49). The above studies show that the pathophysiological outcome of experimental exposure to BPA varies with routes, concentration and dose, cell culture systems, and organisms. Since, the majority of these studies are performed on experimental animals or are cell-culture based studies, it is likely that there is some heterogeneity between the effects of BPA in the different experimental designs and organisms studied.

### BPA effects on allergic and autoimmune conditions

Different studies have reported BPA altered Th1, Th2, and Tregs profile. Majority of these studies have demonstrated augmentation of Th1 type response in BPA-exposed subjects (35, 50–52). Offsprings of C57B6/129svj mice exposed to BPA during gestation have altered cytokine profile with increased Th1 profile and skewing toward Th17 responses (53). Maternal exposure to environmentally relevant BPA dose results in decreased innate immune responses in influenza A-infected adult offspring (54). Perinatal exposure to low doses of BPA from 15th day of pregnancy to pups weaning age, increased anti-ovalbumin (OVA) IgG titers in OVA-tolerized rats accompanied with increased activation of T cells and IFNγ secretion. Oral OVA challenge in these mice increased colonic inflammation, neutrophil infiltration; IFNγ and decreased TGFβ suggesting perinatal exposure of BPA in low doses can make neonatal immune system susceptible to food intolerance (55). BPA exposure via *in utero* and through breast milk promotes development of allergic asthma in BALB/c mice pups sensitized to low dose of OVA (56). However, postnatal BPA exposure alone (through breast milk) was not sufficient to induce allergy in the mouse pups indicating the importance of identification of critical period of BPA exposure in studying different inflammatory conditions (56). In contrast, a recent study showed that BPA intake during pregnancy and breastfeeding or orally did not have significant effect in murine offsprings in experimental allergic asthma (57) and inflammatory bowel disease (IBD) (58).

Contrasting effect of perinatal exposure BPA in different mouse models of MS, experimental autoimmune encephalomyelitis (EAE) disease expression has also been recently reported (59, 60). In Theiler’s-virus induced demyelination, perinatal BPA exposure resulted in decreased anti-viral antibodies, increased onset of disease, and increased inflammation in spinal cord and digestive tract (60). Whereas, in EAE models, C57BL/6J mice (chronic progressive) and SJL/J mice (relapsing-remitting), prenatal BPA exposure did not have significant effect on EAE disease severity and progression (59). These reports suggest that biological effects of BPA exposure vary with age, gender, route and dose, and model of disease. These are important variables to consider when studying immunomodulatory role of BPA.

There was increased autoantibody production by B1 cells from BPA-treated BWF1 mice, a murine model of systemic lupus erythematous (SLE) (61). However, other studies have demonstrated that BPA-fed NZB X NZW F(1) mice had late onset of proteinuria and lowered IFNγ and IL-10 production suggesting protective effect of BPA on SLE (62). Trans-maternal BPA exposure increased diabetes type-1 development in the NOD mice offspring by increasing apoptotic cells and Tregs and decreasing resident macrophages in islets and by inducing systemic immune changes including altered cytokine production (63). Together these studies indicate the importance of critical windows of exposure since early exposure to BPA has modulatory effects on the immune system later in life.

## BPA an Epigenetic Regulator

An important mechanism by which environmental agents can modify gene expression is through altering epigenetics, DNA methylation, histone modification, and microRNA (miRNA). In this regard, several recent studies have confirmed epigenetic regulation by BPA, although most of these studies have focused on epigenetic regulation in non-lymphoid tissues.

### BPA and DNA methylation

Long-lasting effects of perinatal and trans-generational BPA exposure suggest the potential disruption of epigenetic programing of gene expression critical in development, cancer, behavioral, ovarian, and other reproductive functions (64–67). Studies have demonstrated that BPA exposure results in alteration in DNA methylation and expression of specific genes (68). Whether these BPA-mediated alteration in methylation are related to its estrogenic activity remains still unclear. However, a recent study has demonstrated that BPA regulation of methylation was mediated through BPA-ERα regulation of DNA methyltransferase (DNMT)-1 and DNMT-3a expression (Figure 1) (64). BPA exposure of neonatal male rats increased DNMT-3a and -3b expression and also increased methylation at promoter region of ERα and ERβ in testis (69). Different independent groups have also reported BPA-mediated alteration in DNMTs and methyl-CpG binding protein 2 (MECP2) levels and in genome-wide methylation level (70–73). Exposure of BPA promotes global and cytochrome P450 aromatase (cyp19a1a gene-specific) methylation in gonads of adult rare minnow *Gobiocypris rarus* (74). This was also associated with alteration of DNMT mRNA levels. In ovaries, the methylation levels at four CpGs at the 5′ flanking region of cyp19a1a varied with the time of BPA exposure; suppression by 7 days and augmentation by 35 days of BPA exposure (43).

In a recent cross-sectional epidemiological study of pre-pubescent girls in Egypt, it was found that BPA exposure resulted in alteration of methylation profiles. Higher urinary BPA levels were associated with hypomethylation of CpG islands on the X-chromosome and lowered methylation of genes involved in immune function, transport activity, metabolism, and caspase activity (75). Further, it has been found that there is hypomethylation of long interspersed nucleotide elements (LINE 1) in sperms of men exposed to BPA when compared to control group indicating potential of BPA in epigenetic reprograming (76). Non-monotonic dose-dependent effects of DNA methylation patterns were observed in mouse liver following perinatal BPA exposure (77). There was enrichment of regions of altered methylation (RAMs) within CpG island shores (77).

Bisphenol A exposure of F(0) pregnant rats during gestation and lactation, resulted in decreased global DNA methylation in F(1) offspring sperms. In addition, glucokinase (Gck) promoter was completely methylated in F(2) offspring hepatic tissue. There were five unmethylated sites in control offspring indicating maternal BPA exposure can have multigenerational effects on glucose metabolism (78). BPA- and DES-induced antisense transcript, long non-coding RNA HOTAIR in breast cancer cells and in mammary gland of rats, which was mediated by ER-ERE pathway and by chromatin modification (histone methylation and acetylation) (79). BPA increases the expression of Enhancer of Zeste Homolog 2 (EZH2), a histone methyl transferase, in breast cancer cell line (79, 80). Furthermore, EZH2-regulated histone H3 trimethylation was also increased in MCF-7 cell line and in mammary glands of mice exposed to BPA *in utero* (80). *In utero* BPA exposure decreased the expression of phase I and II xenobiotic metabolizing enzyme (XME) genes. This was associated with increased site-specific methylation at COMT and increased average methylation at SULT2A1 promoters (81).

#### BPA Epigenetic Modulation of Gamete, Embryo and Placenta

Epigenetic trans-generational inheritance (ETI) or Germline transmission of epigenetic information between generation is modulated by different environmental stimuli including BPA. These epimutations are changes in methylation and histone modification in germ line and are passed on to subsequent generations (82). Imprinted genes are regulated by differential DNA methylation. Maternal BPA exposure during late stages of oocyte development and early embryonic stages significantly reduced genome-wide methylation levels in placenta and altered methylation of differentially methylated regions (DMRs) such as Snrpn imprinting control region (ICR) and Igf2 DMR1 (83). Low-dose BPA exposure during follicle culture from preantral to antral stage resulted in significant increase in allele methylation errors in DMRs of maternally imprinted genes (Snrpn, Igf2r, and Mest) and decreased in histone H3K9 trimethylation and interkinetochore distance and epigenetic changes in germinal vesicle and metaphase II oocyte, which potentially contribute to chromosome congression failure, meiotic errors, and overall health of offspring (84). BPA exposure of neonatal male rats (F0) resulted in significant hypomethylation at the H19 ICR in sperms of F(0) and in resorbed embryo (F1) (85). In addition, there were decreased Igf2 and H19 mRNA levels in BPA resorbed embryo (F1) compared to viable control embryo. Murine N2A cells exposed to BPA had modest decrease in global DNA methylation accompanied with increased adipocyte differentiation (86). Together, these studies indicate that BPA exposure epigenetically modulates gametes, embryo and placenta, which potentially results in defects in fetal and postnatal development.

Bisphenol A decreased methylation upstream of Agouti gene in viable yellow agouti [A(vy)] mice (87, 88). In BPA-exposed human mammary epithelial cells (HMEC), there was hypermethylation of genes related to the development of most or all tumor types indicating modulatory effect of BPA on HMEC proliferation and senescence (89). *In utero* BPA exposure decreased methylation at Hoxa10 promoter, resulting in increased ERα binding to Hoxa10 ERE thereby increasing ERE-driven Hoxa10 expression (90). These epigenetic modifications result in alteration in ERE sensitivity of different genes and could possibly be a general mechanism of BPA-mediated gene expression.

### BPA and MicroRNAs

Different studies have demonstrated that BPA exposure results in aberrant miRNA expression profile. These miRNAs are believed to target gonadal differentiation, folliculogenesis, and insulin homeostasis (91, 92). Estrogen, BPA, and DDT similarly altered the expression of multiple miRNAs including miR-21 in ER(+) and hormone sensitive MCF-7 breast cancer cell line (93). Placental cell line exposed to BPA had significantly increased miR-146a levels (94). BPA upregulated the expression of oncogenic miR-19a and miR-19b accompanied with the downregulation of miR-19-related downstream proteins such as PTEN, p-AKT, p-MDM2, p53, and proliferating cell nuclear antigen (78). Interestingly, curcumin, which is clinically used for cancer treatment modulated miR-19/PTEN/AKT/p53 axis to protect against BPA-associated breast cancer promotion (95). BPA treatment decreased miR-134 and upregulated the expression of miR-134 targets including pluripotency markers (Oct4, Sox2, and Nanog) in embryonic stem cells (ESC) and embryoid bodies (EB) (96).

### BPA and histone acetylation

Long-term BPA exposure increased *N*-methyl-d-aspartic acid (NMDA) receptor levels and enhanced the expression and function of histone deacetylase 2 (HDAC2) in hippocampus of adult mice (97). Prenatal exposure of Wistar-Furth rats to BPA resulted in increased pro-activation histone H3K4 trimethylation at the promoter region of alpha lactalbumin gene at postnatal day 4 (PND4) and increased alpha lactalbumin mRNA expression in mammary gland (98). Interestingly, majority of differences in gene expression in BPA vs. vehicle-treated group were evident at later stage of life (PND50). These results indicate that fetal BPA exposure can modify postnatal and adult mammary gland epigenome and gene expression, which may contribute to development of pre-neoplastic and neoplastic lesions in adult rat mammary gland (98). To date, there is no information on BPA effects on methylation, miRNAs, and histone acetylation in specific immune subsets.

## Conclusion

Bisphenol A, a model EDC has been shown to modulate not only reproductive and non-lymphoid systems, but it can also affect the immune system. One potential mechanism by which EDC can physiologically affect tissue functioning is by epigenetically regulating ER-targeted genes. While there are only limited studies on epigenetic regulation of BPA on the immune system, studies on many non-lymphoid tissues clearly demonstrate that BPA can alter methylation, histone modification, and miRNAs. Together by integrating current knowledge of trans-generational effect of EDCs on developmental biology, immune function, and epigenetic regulation, it is imperative to design thorough systematic and comprehensive studies to further define the dose, route, and critical windows of exposure of these EDCs and their effects on different chronic inflammatory conditions. A better understanding of BPA regulation of epigenetic mechanism will add to our current understanding about estrogens and their potential contribution in etiopathogenesis in immune-altered states including autoimmune diseases. By extrapolation, it is likely that other EDCs may also epigenetically regulate immune cells, which may have implications in immune conditions including response to infectious agents or susceptibility to autoimmune diseases. There is a distinct gap in this area that warrants studies. These studies will help in establishing safe levels of EDCs in environment especially during the vulnerable fetal life, during which critical immunological education occurs.

## Author Contributions

DK and SAA designed the work, drafted and revised the work and finally approved the version to be published and agree to be accountable for all aspects of the work.

## Conflict of Interest Statement

The authors declare that the research was conducted in the absence of any commercial or financial relationships that could be construed as a potential conflict of interest.

## Abbrevations

AKT, protein kinase B (PKB); COMT, catechol-*O*-methyltransferase; CXCL, chemokine (C-X-C motif) ligand; DPPIII, dipeptidyl-peptidase 3; ERE, estrogen response element; Gata, GATA family transcription factor; IFN, interferon; Igf2r, insulin-like growth factor 2 receptor; IL, interleukin; iNOS, inducible nitric oxide synthase; IRAK, interleukin-1 receptor-associated kinase; Mest, mesoderm-specific transcript homolog protein; MyD88, myeloid differentiation primary response gene 88; Oct4, octamer-binding transcription factor 4; PTEN, phosphatase and tensin homolog; Slc22a18, solute carrier family 22, member 18; Snrpn, small nuclear ribonucleoprotein particles; Sox, SRY (sex determining region Y)-box; SULT2A1, sulfotransferase family, cytosolic, 2A; TNF, tumor necrosis factor; TRAF, TNF receptor associated factor; TRIF, TIR-domain-containing adapter-inducing interferon-β.
